# Benchmarking antibody modeling tools across structure prediction, docking, and paratope–epitope interface analysis

**DOI:** 10.1093/bioadv/vbag223

**Published:** 2026-08-11

**Authors:** Zeyuan Yu, Jilei Wu, Ziyao Ning, Chunxia Qiao, Jing Wang, Xinying Li, Chenghua Liu, Guojiang Chen, Jiannan Feng, Jijun Yu

**Affiliations:** School of Artificial Intelligence and Data Science, University of Science and Technology of China, Hefei, Anhui 230026, China; College of Biotechnology, Jiangsu University of Science and Technology, Zhenjiang, Jiangsu 212100, China; School of Pharmacy, Qingdao University, Qingdao, Shandong 266071, China; State Key Laboratory of National Security Specially Needed Medicines, Beijing 100000, China; State Key Laboratory of National Security Specially Needed Medicines, Beijing 100000, China; State Key Laboratory of National Security Specially Needed Medicines, Beijing 100000, China; State Key Laboratory of National Security Specially Needed Medicines, Beijing 100000, China; State Key Laboratory of National Security Specially Needed Medicines, Beijing 100000, China; State Key Laboratory of National Security Specially Needed Medicines, Beijing 100000, China; State Key Laboratory of National Security Specially Needed Medicines, Beijing 100000, China

## Abstract

**Motivation:**

Computational antibody engineering requires reliable prediction of antibody variable-fragment structures, antigen–antibody complexes, and binding interfaces. However, publicly available tools for these tasks have rarely been compared across the complete workflow under a controlled and statistically grounded design.

**Results:**

We evaluated ImmuneBuilder, IgFold, AlphaFold3, GRAMM, and dyMEAN on 50 non-redundant humanized antibody–antigen complexes using multiple retained predictions and paired statistical testing. All three antibody structure predictors were accurate, with AlphaFold3 performing best overall and for the third complementarity-determining region of the heavy chain. AlphaFold3 also substantially outperformed GRAMM and dyMEAN in complex prediction, producing medium- or high-quality binding interfaces for 46% of the complexes, although overall interface accuracy remained limited. When docking was reliable, AlphaFold3 accurately recovered epitope and paratope residues, salt bridges, and non-bonded contacts, but reproduced hydrogen bonds and fine-grained contact strengths less consistently. These findings provide practical guidance for selecting tools across antibody-modeling workflows and identify persistent limitations in fine-grained interface prediction.

**Availability and implementation:**

Data, structural predictions, evaluation results, and analysis code are available from Zenodo under record 20710876.

## 1 Introduction

Antibody-based therapeutics are widely used in the treatment of cancer (Sliwkowski and Mellman 2013) and autoimmune diseases (Chan and Carter 2010). Traditional antibody structural characterization relies on experimental techniques such as X-ray crystallography and cryo-electron microscopy (Chiu *et al*. 2019), which are accurate but time-consuming and costly, and therefore less suited to large-scale or rapid structure characterization.

Recent advances in computational structural biology, particularly deep learning–based protein structure prediction, have enabled new strategies for antibody engineering. AlphaFold2 (Jumper *et al*. 2021) marked a paradigm shift in protein structure prediction, achieving accuracy approaching experimental resolution for many protein targets. AlphaFold-derived structural models have since been used in downstream tasks such as protein classification, signal/transit peptide prediction, and protein–ligand interaction analysis (Escobedo *et al*. 2024, Pei *et al*. 2024, Sanaboyana and Elcock 2024). Recent studies have further evaluated AlphaFold-family methods for biomolecular-complex and ligand-bound structure prediction (He *et al*. 2025). In antibody modeling, however, specialized studies continue to highlight persistent limitations, particularly for CDR loop conformations and antibody model quality (Fernández-Quintero *et al*. 2023, Chen *et al*. 2024). These challenges have driven the development of specialized deep-learning-based tools for antibody modeling, such as ABlooper (Abanades *et al*. 2022) and IgFold (Ruffolo *et al*. 2023). Predicting antibody structures, modeling antigen–antibody complexes, and analyzing the paratope–epitope interface are key steps in structure-based antibody engineering (Joubbi *et al*. 2024, Santuari *et al*. 2024).

Accurate prediction of the antibody fragment variable (Fv) region, particularly the CDRs, is a prerequisite for modeling antibody–antigen complexes. Current AI-assisted antibody structure prediction methods include antibody-specific tools, such as ABodyBuilder (Leem *et al*. 2016), ImmuneBuilder/ABodyBuilder2 (Abanades *et al*. 2023), and IgFold (Ruffolo *et al*. 2023), as well as general-purpose structure predictors represented by AlphaFold3 (AF3) (Abramson *et al*. 2024). Antibody-specific methods incorporate antibody-focused structural priors or domain-specific training to model variable regions and flexible CDR loops (Abanades *et al*. 2023, Ruffolo *et al*. 2023, Chen *et al*. 2024), whereas AF3 uses a diffusion-based architecture for general biomolecular complex prediction and builds on AlphaFold2 (Jumper *et al*. 2021) and AlphaFold-Multimer (Evans *et al*. 2021). Because these methods follow distinct modeling strategies, a controlled comparison of their performance provides practical guidance for tool selection in antibody-modeling workflows.

Accurate prediction of antibody–antigen complex structures remains a key challenge in computational antibody modeling. Current approaches fall broadly into two groups: traditional physics- or geometry-based docking algorithms and AI-driven deep learning models. Conventional tools such as GRAMM (Singh *et al*. 2024) estimate stable conformations from shape complementarity and intermolecular energy landscapes, but rigid-body docking approaches can be limited when substantial conformational flexibility or induced-fit changes occur at protein–protein interfaces (Desta *et al*. 2020). dyMEAN (Kong *et al*. 2023), a deep learning method, applies dynamic multi-channel equivariant graph networks to generate full-atom antibody structures and model antibody–antigen complexes using epitope information and shadow-paratope modeling. AF3 (Abramson *et al*. 2024) is a general-purpose complex predictor that uses a diffusion-based framework to jointly model proteins and other biomolecular components.

More recently, DeepSCFold (Hou *et al*. 2025) was developed to improve protein-complex structure modeling by using sequence-derived structural-complementarity information to construct paired multiple-sequence alignments. On a temporally separated benchmark of 89 antibody–antigen complexes, DeepSCFold was reported to achieve competitive interface DockQ relative to AF3 and a 12.4% improvement in prediction success rate at DockQ > 0.23, highlighting the rapid evolution of immune-complex prediction methods.

Despite these developments, these tools have rarely been compared directly under a controlled, statistically grounded setup, and their influence on downstream paratope–epitope analysis remains unclear. A practical, like-for-like comparison is therefore needed to guide tool selection in antibody-modeling workflows.

**Figure 1:**
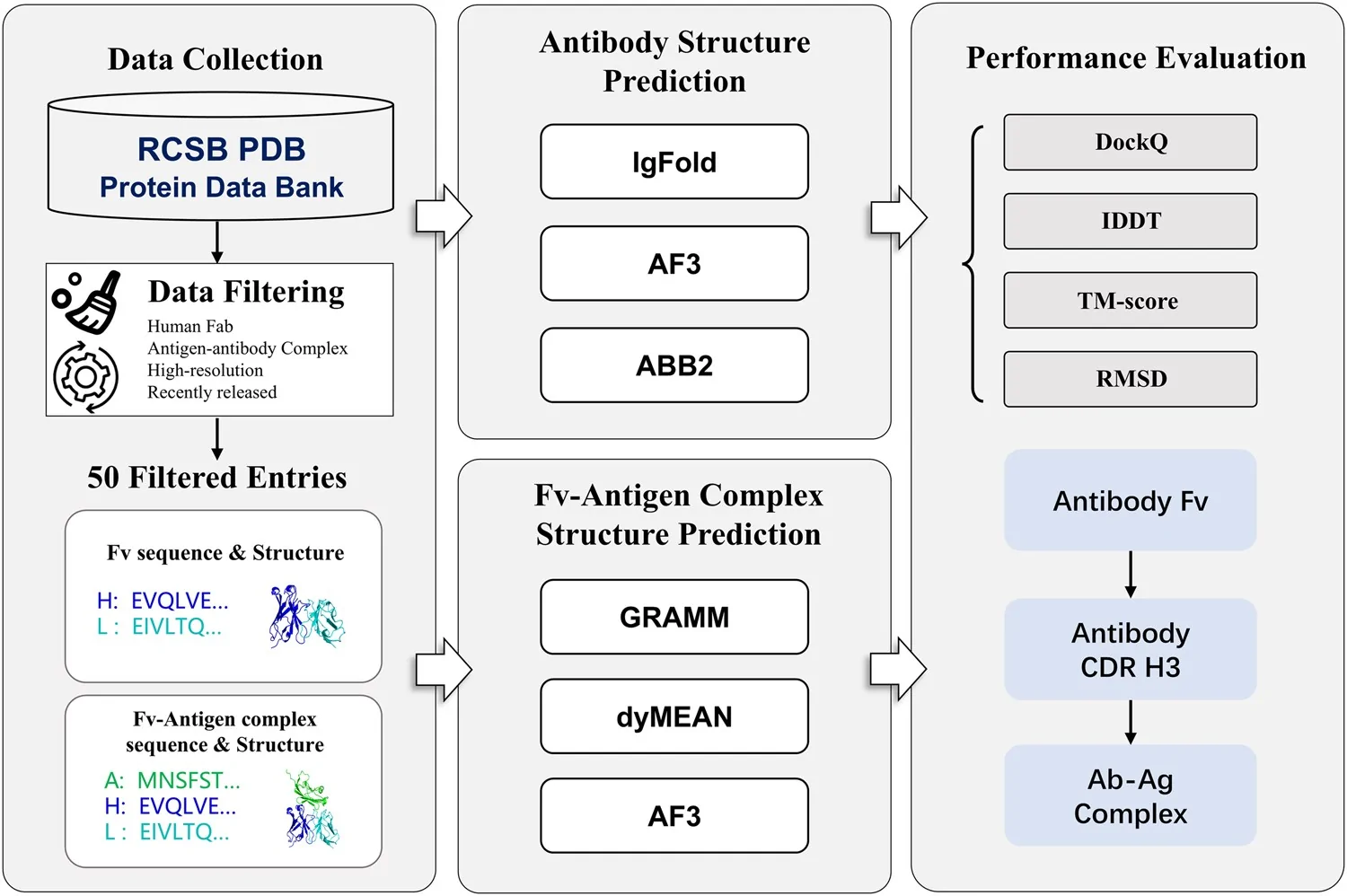
Overview of the evaluation workflow. Five widely used publicly available tools were evaluated on a non-redundant set of 50 humanized antibody–antigen complexes from the PDB, using four structural metrics (RMSD, lDDT, TM-score, DockQ) for Fv and complex prediction, followed by paratope–epitope interface recovery analysis on complexes with reliable docking poses. Abbreviations: AF3, AlphaFold3; ABB2, ABodyBuilder2; Fv, fragment variable; CDR, complementarity-determining region.

Paratope–epitope analysis depends on the predicted complex structure and is therefore tightly coupled to docking accuracy (Joubbi *et al*. 2024). Approximate spatial alignment of the chains does not by itself guarantee a faithful binding interface, so interface quality should be assessed jointly with docking accuracy using quantitative interface-level information, including interface residues, hydrogen bonds, salt bridges, and non-bonded contacts derived from structural interaction analysis tools such as PDBsum (Laskowski 2001). Accordingly, in this study we first evaluated Fv- and complex-level structural accuracy using four widely adopted metrics, including RMSD, lDDT, TM-score, and DockQ, and then assessed paratope–epitope interface recovery for complexes with reliable docking poses (Fig. 1). All comparisons were performed on a non-redundant set of humanized antibody–antigen complexes using multiple prediction outputs and paired statistical testing, providing a more reliable basis for examining antigen–antibody recognition.

## 2 Methods

### 2.1 Dataset

We assembled a benchmark set of 50 humanized antibody–antigen Fab complexes from the Protein Data Bank (PDB) (Berman *et al*. 2000). To avoid overlap with the training data of the evaluated models, we restricted the set to structures released after the cutoff date of the most recent method (AF3; 19 December 2023), and required a resolution better than 3.0 Å to ensure structural reliability. The deposition date and resolution of every entry are reported in Supplementary Data 1, available at supplementary material  *Bioinformatics Advances* online.

To characterize sequence redundancy, we clustered the 50 antibodies at the heavy-chain, light-chain, paired-Fv, and CDR-H3 levels using MMseqs2 (Steinegger and Söding 2017) at 90% and 95% sequence-identity thresholds. The set was essentially non-redundant at the determinant levels: at both thresholds, every CDR-H3 sequence—and, at 95% identity, every paired-Fv sequence—formed its own cluster, and the heavy chains yielded 49–50 clusters. Only the light chains showed mild redundancy, forming 43–46 clusters, consistent with the limited germline diversity of antibody light chains. Full per-level clustering results are provided in Table S1 and Supplementary Data 2, available at supplementary material  *Bioinformatics Advances* online.

### 2.2 Evaluation metrics

To compare predicted structures with their experimental references, we employed four widely used structural evaluation metrics.

#### 2.2.1 TM-score (template modeling score)

TM-score (Zhang and Skolnick 2004) ranges from 0 to 1 and quantifies global structural similarity between two protein structures. Values closer to 1 indicate high topological agreement. Unlike RMSD, TM-score is length-normalized and is therefore more robust when comparing proteins of different sizes. Higher TM-scores reflect closer overall folding similarity.

#### 2.2.2 RMSD (root mean square deviation)

RMSD measures the average distance between corresponding aligned atoms, typically Cα atoms. Lower RMSD values indicate smaller geometric deviations. In many protein-modeling tasks, RMSD values below approximately 2 Å are often associated with near-native similarity, although the appropriate threshold can vary depending on structural flexibility and task context.

#### 2.2.3 lDDT (local distance difference test)

lDDT (Mariani *et al*. 2013) is a superposition-free local accuracy metric ranging from 0 to 1 that evaluates how well local atomic distances are preserved. Higher scores indicate greater residue-level agreement, making lDDT particularly suited for identifying local inaccuracies even when global alignment differs.

#### 2.2.4 DockQ

DockQ (Basu and Wallner 2016) is a composite score ranging from 0 to 1 for protein–protein complex models, combining interface RMSD, ligand RMSD, and the fraction of native contacts, with higher values indicating more near-native interfacial geometry. For Fv prediction, DockQ was computed for the heavy–light variable-domain interface. For complex prediction, because an antigen–antibody complex contains two distinct antibody chains, we computed DockQ separately for the heavy chain–antigen and light chain–antigen interfaces and report the two values independently rather than as a single averaged score.

### 2.3 Evaluation and visualization tools

A suite of established computational tools and online services was employed to perform structural evaluation, molecular interaction analysis, and visualization throughout this study.

#### 2.3.1 PyMOL

PyMOL (Schrödinger, LLC, 2024) version 3.0.5 was used for macromolecular visualization, inter-chain and inter-structure superposition, and inspection of atomic-level interactions, including hydrogen bonds and salt bridges.

#### 2.3.2 Structure assessment online service

The Structure Assessment Online Service (Waterhouse *et al*. 2024) was used as a reference tool for evaluating structural similarity metrics, including TM-score, lDDT, RMSD, and DockQ, and for preliminary validation and visualization of structure-evaluation results.

#### 2.3.3 PDBsum

PDBsum (Laskowski 2001) was employed for detailed interface analysis, providing residue–residue interaction maps and graphical summaries describing antibody–antigen contact patterns.

#### 2.3.4 AbYsis

The AbYsis database and online service (Swindells *et al*. 2017) were used to identify complementarity-determining regions (CDRs). Throughout this study, we adopted the IMGT definitions for CDR and framework-region boundaries.

### 2.4 Publicly available tools for antibody Fv-region and antibody–antigen complex structure prediction

#### 2.4.1 ABB2

**Figure 2:**
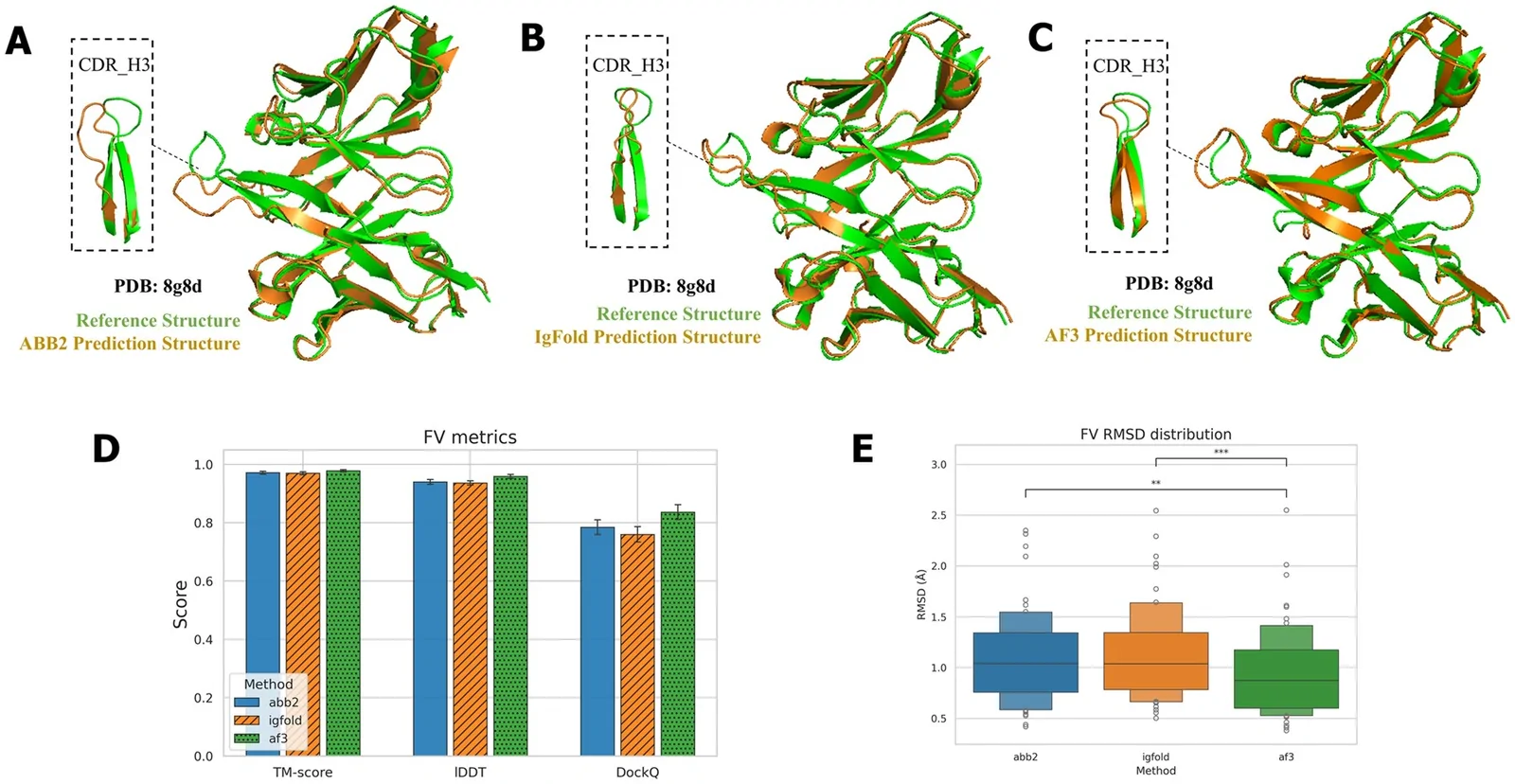
Visualization and quantitative evaluation of antibody Fv structure predictions by ABB2, IgFold, and AlphaFold3. (A–C) Structural comparison of one representative antibody (PDB ID: 8g8d) predicted by ABB2, IgFold, and AlphaFold3, shown alongside the reference structure, with a detailed view of the CDR-H3 loop. (D) Mean performance across the 50 antibodies for three metrics (TM-score, lDDT, and DockQ) over the Fv region; bars and error bars denote the mean and its bootstrap 95% confidence interval, with the five predictions per antibody aggregated per target. (E) Distribution of Fv RMSD across the 50 antibodies for the three methods. Pairwise comparisons used the paired Wilcoxon signed-rank test with Benjamini–Hochberg correction. Complete per-antibody results are provided in Supplementary Data 3, available at supplementary material  *Bioinformatics Advances* online.

ImmuneBuilder includes the ABodyBuilder2 (ABB2) module for antibody structure prediction (Abanades *et al*. 2023). ABB2 is based on a modified AlphaFold-Multimer backbone (Evans *et al.* 2021) and uses iterative deep-learning modules, including invariant point attention, to update structural representations and model antibody-specific conformational features. Full-atom antibody structures are reconstructed from predicted geometric parameters. We deployed the ABB2 code and parameters locally using four RTX 3080 GPUs with 10 GB memory. The input consisted of antibody heavy- and light-chain sequences, and the output was a predicted Fv-region PDB file.

#### 2.4.2 IgFold

IgFold is an antibody structure prediction tool that predicts antibody variable-region structures directly from heavy- and light-chain sequences (Ruffolo *et al*. 2023). It uses representations from the antibody language model AntiBERTy (Ruffolo *et al.* 2021), followed by graph-based structural modules and invariant point attention to generate physically plausible antibody structures. We deployed the IgFold code and parameters locally using four RTX 3080 GPUs with 10 GB memory. The input consisted of antibody heavy- and light-chain sequences, and the output was a predicted Fv-region PDB file.

#### 2.4.3 AlphaFold3

AF3 (Abramson *et al*. 2024) is a general-purpose biomolecular complex predictor. It integrates sequence and interaction information through Pairformer-based representations and uses a diffusion-based structure-generation framework (Ho *et al*. 2020) to model biomolecular structures and complexes. We used the official AlphaFold3 web server. The inputs were antibody heavy- and light-chain sequences for Fv prediction, or antibody and antigen sequences for complex prediction; the output was a predicted PDB structure or complex model.

#### 2.4.4 GRAMM

GRAMM (Singh *et al*. 2024) is a classical rigid-body docking method for protein-complex prediction. We used the public GRAMM web server in free docking mode, in which rigid-body translational and rotational searches are used to generate candidate antigen–antibody docking poses. For each target, the top five docking poses returned by the server were retained for evaluation. The input consisted of antigen and antibody PDB structures, and the output was a PDB file of the predicted docking pose.

#### 2.4.5 DyMEAN

DyMEAN (Kong *et al*. 2023) is a deep-learning method for antibody structure generation and antigen–antibody complex modeling. In the complex-modeling setting, dyMEAN uses epitope information and a shadow-paratope representation to guide antibody–antigen docking. It employs E(3)-equivariant neural-network operations to maintain geometric consistency (Satorras *et al*. 2021). Candidate poses are generated by aligning predicted interface representations to antigen epitope features, including use of the Kabsch algorithm for coordinate alignment (Kabsch 1976). The antigen epitope information used for dyMEAN input was obtained from HDOCK-based docking results (Yan *et al*. 2020). We deployed dyMEAN code and parameters locally using four RTX 3080 GPUs with 10 GB memory. The inputs were the antigen PDB structure, antibody heavy- and light-chain sequences, and antigen epitope information; the output was a predicted antibody–antigen complex PDB file.

#### 2.4.6 Prediction generation and configuration

For each tool and each target, five predicted structures or docking poses were retained to assess prediction variability and robustness, yielding 250 predictions per task across the 50 complexes. For AlphaFold3, five server-generated models returned by the official AlphaFold3 server were collected for each antibody–antigen complex. For GRAMM, the top five docking poses returned by the public GRAMM web server were retained. For ABB2/ImmuneBuilder and IgFold, five repeated local prediction runs were performed for each target using different random seeds where seed control was available. For dyMEAN, the sample number parameter was set to 5 during conformational generation.

Locally deployed tools, including ABB2, IgFold, and dyMEAN, were run on four NVIDIA RTX 3080 GPUs with 10 GB memory per GPU, whereas AF3 was accessed through the official AlphaFold3 server and GRAMM through its public web server. Software versions, key run-time parameters, and approximate computational costs for each tool are summarized in Table S2, available at supplementary material  *Bioinformatics Advances* online.

### 2.5 Statistical analysis

Unless otherwise stated, the statistical unit was the individual complex. For each target and tool, the five predictions were summarized by their mean, giving one value per target (*n = *50). Group-level performance was reported as the mean across targets with a 95% confidence interval estimated by bootstrap resampling with 10 000 resamples. Pairwise comparisons between tools for a given metric were performed using two-sided Wilcoxon signed-rank tests on paired per-target values, with Benjamini–Hochberg correction across tool pairs (Benjamini and Hochberg 1995); an adjusted *P* < 0.05 was considered significant. To assess sensitivity to model stochasticity, we additionally computed, for each target, the standard deviation across the five predictions and compared its magnitude with between-tool differences. Per-tool run-to-run variability and the between-tool comparisons are reported in Supplementary Tables S3 and S4, available at supplementary material  *Bioinformatics Advances* online, respectively. Linear relationships, such as those between Fv RMSD and CDR-H3 RMSD, were summarized by ordinary least-squares regression and the coefficient of determination (*R*²). All analyses were performed in Python using NumPy, SciPy, and pandas.

### 2.6 Paratope–epitope interface recovery analysis

To evaluate how faithfully AF3 reconstructs antigen–antibody binding interfaces beyond a single case, we compared predicted and experimental interfaces across all 50 complexes. Inter-chain contacts, including hydrogen bonds, salt bridges, and non-bonded contacts, as well as per-residue-pair atom-contact counts, were extracted from both the reference and AF3-predicted complexes using PDBsum (Laskowski 2001), considering antibody–antigen interfaces only. Because AF3 numbers residues sequentially whereas the reference structures use author or IMGT numbering, reference and predicted residues were mapped to a common per-chain coordinate frame defined by the reference modeled sequence. AF3 chains were assigned to heavy-chain, light-chain, and antigen roles by sequence matching, and this mapping was verified for all 50 complexes.

For each complex and each of the five AF3 predictions, the experimental interface was taken as the set of antibody–antigen residue–residue contacts, and the following quantities were computed relative to it: (i) epitope- and paratope-residue recovery, defined by precision, recall, and F1 score of the antigen-side and antibody-side interface residues, with the antibody side further split into heavy and light chains; (ii) residue-pair contact recovery, defined by precision, recall, and F1 score of contacting residue pairs; (iii) per-interaction-type recovery, defined by recall of hydrogen bonds, salt bridges, and non-bonded contacts; and (iv) contact-multiplicity agreement, defined by the Spearman correlation between reference and predicted per-residue-pair atom-contact counts. Per-complex values were obtained by averaging over the five predictions and summarized with bootstrap 95% confidence intervals as described above. Because interface recovery is informative primarily when the docking pose is approximately correct, we report these metrics both over all 50 complexes and over subsets with reliable or acceptable docking quality, defined by DockQ ≥ 0.49 and DockQ ≥ 0.23, respectively.

## 3 Results

### 3.1 Antibody Fv structure evaluation

To systematically assess antibody structure prediction performance, we focused on the antibody variable fragment (Fv), which encompasses the heavy and light chain variable domains including the critical complementarity-determining regions (CDRs).

We retrieved native antigen-binding fragment (Fab) structures from the Protein Data Bank (PDB) (Berman *et al*. 2000). For the antibody Fv prediction tasks, we extracted the Fv region, which includes both heavy-chain and light-chain variable domains, from the original PDB files, preserving both structural and sequence information. The isolated sequences were then independently processed through three prediction methods (IgFold, ABB2 and AF3), each generating predicted Fv structures. We calculated the root-mean-square deviation (RMSD), template modeling score (TM-score), local Distance Difference Test (lDDT), and DockQ metrics by comparing the predicted structures with PDB experimental references.

We compared each predicted Fv model with its experimental reference at both the global and local levels (Fig. 2). All three methods reconstructed the Fv region with high accuracy: the mean TM-score exceeded 0.97, the mean lDDT exceeded 0.93, and the mean DockQ exceeded 0.76 for every method (Fig. 2D). AF3 achieved the highest value for all three metrics, and these advantages were statistically significant (paired Wilcoxon signed-rank test with Benjamini–Hochberg correction; all adjusted *P* < 0.01 versus both ABB2 and IgFold). Consistent with this, the Fv RMSD distribution (Fig. 2E) was lowest for AF3 (median 0.87 Å) and comparable between ABB2 and IgFold (median 1.04 Å for both), and the majority of predictions fell below 1.4 Å (76%, 76%, and 86% for ABB2, IgFold, and AF3, respectively). All three models therefore produced reliable Fv predictions, with AF3 the most accurate. Nevertheless, close inspection of the CDR-H3 loop in the representative example (Fig. 2A–C) revealed visible deviations, indicating that this region remains the principal challenge and motivating a focused analysis of CDR-H3 accuracy.

**Figure 3:**
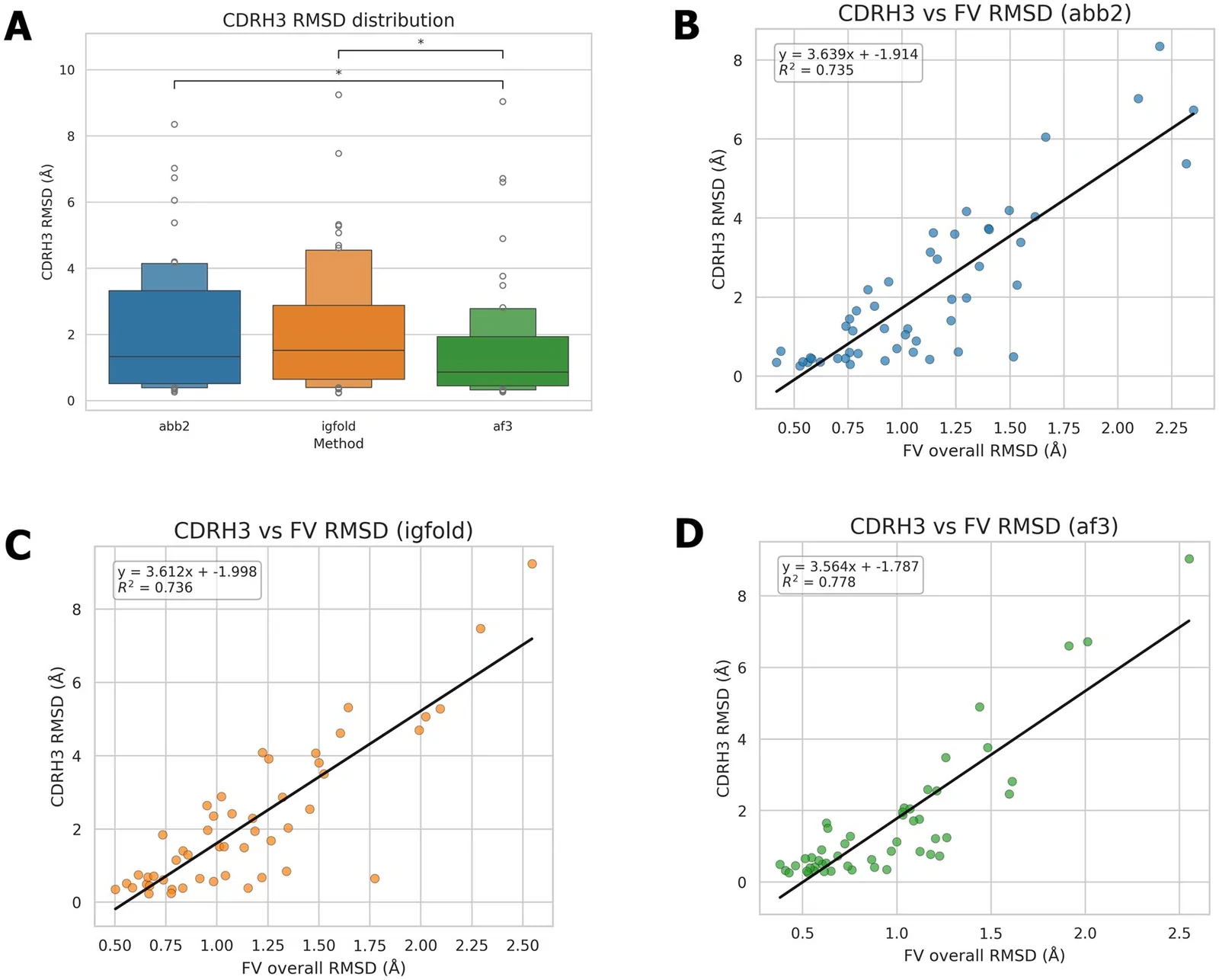
Comparative analysis of CDR-H3 loop accuracy. (A) Distribution of CDR-H3 RMSD across the 50 antibodies for the three methods; significance from paired Wilcoxon signed-rank tests with Benjamini–Hochberg correction (*, adjusted *P* < 0.05; ns, not significant). (B–D) Relationship between the overall Fv RMSD (*x*-axis) and the CDR-H3 RMSD (*y*-axis) for ABB2, IgFold, and AF3, respectively; each point is one antibody (mean of five predictions), and the solid line is the ordinary least-squares fit.

To quantify CDR-H3 accuracy, we computed the CDR-H3 RMSD across the 50 antibodies (Fig. 3A). In contrast to the overall Fv region, the three methods differed significantly in this loop: AF3 achieved the lowest CDR-H3 RMSD (median 0.86 Å), significantly lower than both ABB2 (median 1.34 Å; BH-adjusted *P* = 0.026) and IgFold (median 1.52 Å; adjusted *P* = 0.017), which were statistically indistinguishable from each other (adjusted *P* = 0.75). AF3 also showed the most concentrated distribution (interquartile range 0.45–1.94 Å). Comparing the CDR-H3 RMSD (Fig. 3A) with the overall Fv RMSD (Fig. 2E) shows that errors in this loop are substantially larger and more dispersed than across the Fv as a whole, motivating an analysis of how the two are related (Fig. 3B–D).

The regression analysis (Fig. 3B–D) revealed a strong linear relationship between the overall Fv RMSD and the CDR-H3 RMSD for all three methods (*R*² = 0.735–0.778). AF3 showed the highest coefficient of determination (*R*² = 0.778), indicating the tightest coupling between its global Fv accuracy and its local CDR-H3 accuracy, followed by IgFold (*R*² = 0.736) and ABB2 (*R*² = 0.735). The regression slopes were similar across methods (ABB2, 3.64; IgFold, 3.61; AF3, 3.56), implying that a given increase in overall Fv error corresponds to an approximately 3.6-fold larger increase in CDR-H3 error—consistent with CDR-H3 being the principal contributor to Fv structural deviation.

We next examined run-to-run reproducibility, which is relevant because AF3 is a generative, diffusion-based model. The two antibody-specific tools were essentially deterministic across the five predictions (median within-antibody standard deviation ≤ 0.003 Å for the Fv and ≤ 0.016 Å for CDR-H3), whereas AF3 showed larger but still modest variability (0.064 Å for the Fv and 0.144 Å for CDR-H3) (Table S3, available at supplementary material  *Bioinformatics Advances* online). This run-to-run variability was small relative to the differences between methods (Table S4, available at supplementary material  *Bioinformatics Advances* online), indicating that AF3's accuracy advantage does not arise from favorable seed selection. The maximum per-antibody deviations were comparable across the three tools (Fv ≤ 2.55 Å; CDR-H3 ≤ 9.25 Å), with no method showing systematically larger extremes.

In summary, all three methods reconstruct the overall Fv with high accuracy but differ in detail. AF3 was the most accurate at both the global Fv and the local CDR-H3 level and showed the tightest coupling between the two (*R*² = 0.778), at the cost of slightly higher run-to-run variability than the deterministic antibody-specific tools. In practice, AF3 is preferable when maximal Fv—and especially CDR-H3—accuracy is required, whereas ABB2 and IgFold provide fast, fully reproducible predictions well suited to high-throughput modeling, with otherwise comparable per-antibody accuracy.

### 3.2 Antibody-antigen complex structure evaluation

For the antigen–antibody complex structure prediction tasks, we extracted the structural and sequence information of both the antigen and the antibody Fv region directly from the PDB files. These data were independently provided as inputs to three prediction methods (dyMEAN, GRAMM, and AF3) to generate the corresponding complex structures. The predicted complexes were then compared with the experimental reference structures using RMSD, TM-score, lDDT, and DockQ as quantitative evaluation metrics.

**Figure 4:**
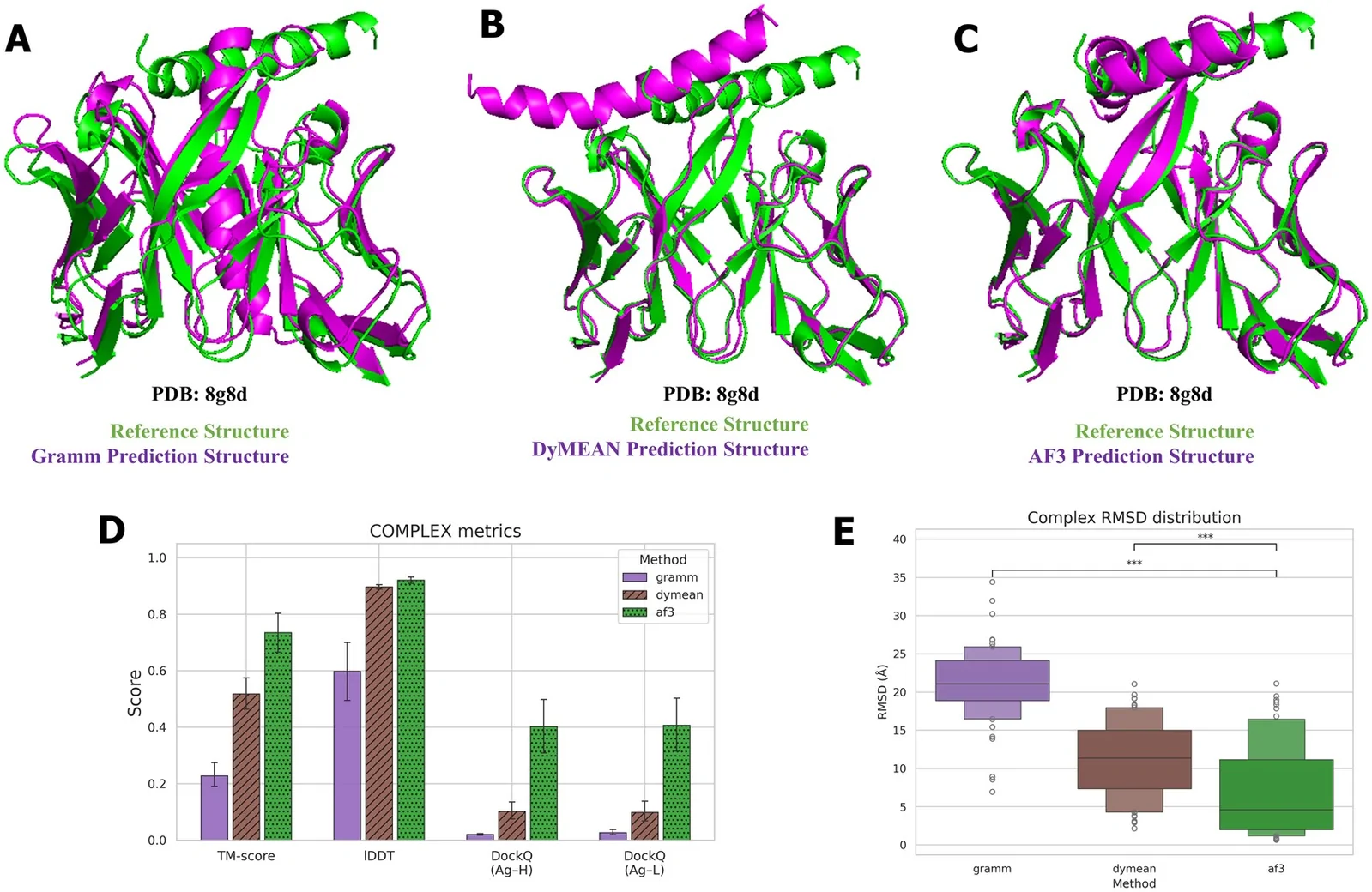
Visualization and quantitative evaluation of antibody–antigen complex structure predictions. (A–C) Structural comparison of one representative complex (PDB ID: 8g8d) predicted by GRAMM, dyMEAN, and AF3, shown alongside the reference complex. (D) Mean performance across the 50 complexes for TM-score, lDDT, and DockQ, the last reported separately for the heavy-chain–antigen (Ag–H) and light-chain–antigen (Ag–L) interfaces; bars and error bars denote the mean and its bootstrap 95% confidence interval. (E) Distribution of complex RMSD across the 50 complexes. Pairwise comparisons used the paired Wilcoxon signed-rank test with Benjamini–Hochberg correction. Complete per-complex results are provided in Supplementary Data 3, available at supplementary material  *Bioinformatics Advances* online.

We summarize a representative complex together with the quantitative results across the 50 complexes (Fig. 4). The structural superpositions (Fig. 4A–C) show that none of the three methods reproduced the binding pose with high accuracy, with clearly visible deviations from the reference. Quantitatively (Fig. 4D), the three methods differed markedly and consistently, AF3 being the most accurate and GRAMM the least. AF3 best preserved the global complex topology (mean TM-score 0.74, mean lDDT 0.92), followed by dyMEAN (0.52 and 0.90) and GRAMM (0.23 and 0.60). The same ordering held, far more steeply, for interface accuracy: the mean DockQ of the heavy- and light-chain–antigen interfaces was 0.40 and 0.41 for AF3, but only 0.10 and 0.10 for dyMEAN and 0.02 and 0.03 for GRAMM (all pairwise differences highly significant; paired Wilcoxon signed-rank test with Benjamini–Hochberg correction, adjusted *P* < 0.001). Thus, even AF3—despite well-preserved global topology—reached only moderate interface accuracy, whereas dyMEAN and GRAMM largely failed to position the antigen correctly.

The complex RMSD distributions (Fig. 4E) reinforced this ordering: AF3 achieved the lowest median RMSD (≈4.6 Å) and the narrowest distribution, dyMEAN was intermediate (≈11 Å), and GRAMM the worst (≈21 Å) (all pairwise differences adjusted *P* < 0.001). Together, these results indicate that, although AF3 clearly outperforms the other two methods, interface accuracy remains limited even for the best method—motivating a closer analysis of the predicted binding interfaces.

**Figure 5:**
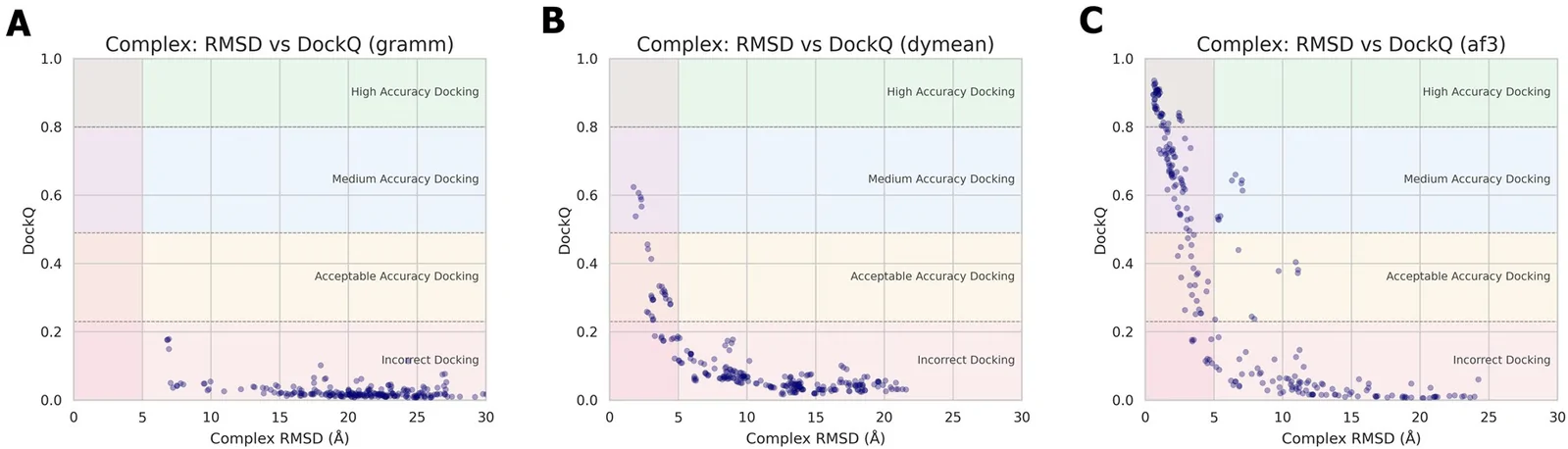
Relationship between complex RMSD and DockQ for antigen–antibody predictions. (A–C) Scatter plots of DockQ (*y*-axis) versus complex RMSD (*x*-axis) for GRAMM, dyMEAN, and AF3, respectively; each point is one prediction (five per complex). Horizontal bands mark the official DockQ quality categories (Incorrect < 0.23; Acceptable 0.23–0.49; Medium 0.49–0.80; High ≥ 0.80), and the shaded vertical region marks complex RMSD < 5 Å.

We analyzed the relationship between RMSD and DockQ for the predicted structures of 50 antigen–antibody complexes, with the results for GRAMM, dyMEAN, and AF3 shown in Fig. 5A–C, respectively. Accurate interface prediction is essential for meaningful antigen–antibody docking, as even slight shifts in the binding site can render the docking pose unreliable. According to the official DockQ classification (Basu and Wallner 2016), predictions are categorized as High (≥ 0.8), Medium (0.49–0.8), Acceptable (0.23–0.49), or Incorrect (< 0.23). Given the stringent accuracy requirements of antigen-antibody interface modeling, we defined predictions with DockQ ≥ 0.49 (Medium or High quality) as reliably aligned to the correct binding region, whereas those below this threshold were considered failures for subsequent analyses.

As shown in Fig. 5A–C, the methods differed sharply in their ability to reach reliable docking. GRAMM produced no prediction of at least medium quality (DockQ ≥ 0.49), and dyMEAN reached medium quality for only a single complex; neither produced any high-quality prediction (DockQ ≥ 0.80), and their points clustered near DockQ = 0 (Fig. 5A and B). In contrast, AF3 reached at least medium quality for 23 of the 50 complexes (46%), including 8 high-quality predictions (16%), and these successful cases clustered at complex RMSD < 5 Å (Fig. 5C). Accordingly, the subsequent interface analysis was restricted to the AF3 predictions.

### 3.3 Paratope-epitope interaction analysis

**Figure 6:**
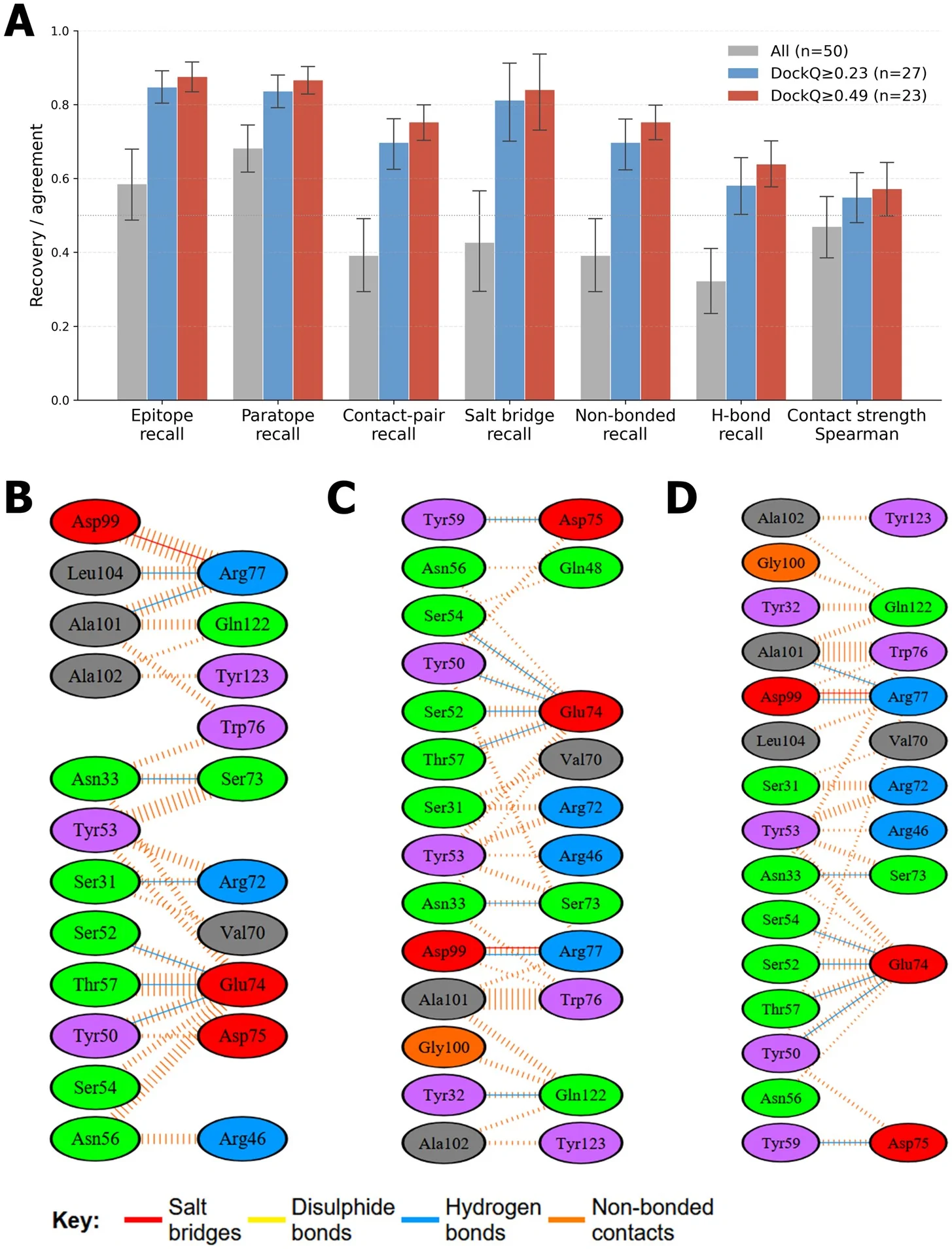
Paratope–epitope interface recovery by AlphaFold3. (A) Interface-recovery metrics for AF3 predictions relative to the experimental interface, summarized across three subsets of complexes: all 50 complexes (grey), complexes with at least acceptable docking (DockQ ≥ 0.23, *n = *27; blue), and complexes with at least medium-quality docking (DockQ ≥ 0.49, *n = *23; red). Bars show, from left to right, the recall of epitope residues, paratope residues, residue–residue contact pairs, salt bridges, non-bonded contacts, and hydrogen bonds, together with the Spearman correlation between predicted and experimental per-residue-pair atom-contact counts (contact strength). Bars and error bars denote the mean and its bootstrap 95% confidence interval. Full precision, recall, and F1 values for all subsets are given in Table S5, available at supplementary material  *Bioinformatics Advances* online. (B–D) PDBsum-style diagrams of the antibody–antigen interface for one representative reliably docked complex (PDB ID: 9cf6): (B) the experimental reference interface and (C, D) two of the five AlphaFold3 predictions. In each panel, heavy-chain (paratope) residues are shown on the left and antigen (epitope) residues on the right; lines denote interfacial interactions colored by type (red, salt bridges; yellow, disulphide bonds; blue, hydrogen bonds; orange, non-bonded contacts).

Having established that AF3 is the only method to dock a substantial fraction of complexes correctly, we next asked how faithfully it reconstructs the binding interface itself. Because interface accuracy is meaningful only when the overall pose is approximately correct, we quantified interface recovery across all 50 complexes and stratified the results by docking quality (Methods; Fig. 6). Interface recovery was strongly gated by docking success: when docking failed (DockQ < 0.23), the predicted and experimental contacts barely overlapped (mean residue-pair F1 = 0.04), whereas for complexes that reached at least medium quality (DockQ ≥ 0.49), recovery was high (mean residue-pair F1 = 0.74). The following analysis therefore focuses on the 23 reliably docked complexes; recovery for all three docking-quality strata is shown in Fig. 6A.

Within these complexes, AF3 identified the interface residues with high accuracy. It recovered epitope residues with a mean precision of 0.87 and recall of 0.88 (F1 = 0.87; 95% CI 0.84–0.89) and paratope residues with a precision of 0.85 and recall of 0.87 (F1 = 0.85; 95% CI 0.82–0.88). Paratope recovery was slightly higher for the heavy chain than for the light chain (F1 = 0.86 versus 0.82), consistent with the dominant contribution of the heavy chain—and particularly CDR-H3—to antigen binding. Thus, for correctly docked complexes, AF3 reliably pinpoints which residues form the antigen–antibody interface on both partners.

At the finer level of individual contacts, recovery was good but more variable. AF3 reproduced about three-quarters of the experimental residue–residue contacts (recall 0.75; F1 0.74). Resolving contacts by chemical type revealed a clear hierarchy: salt bridges were recovered most reliably (recall 0.84; 95% CI 0.73–0.94, among the 17 complexes containing interfacial salt bridges), non-bonded contacts almost as well (recall 0.75), whereas hydrogen bonds were recovered substantially less often (recall 0.64; 95% CI 0.58–0.70). In addition, the strength of individual contacts—quantified as the number of atom–atom contacts per residue pair—was only moderately correlated between prediction and experiment (Spearman *ρ* = 0.57). AF3 therefore captures the location and the strong electrostatic features of the interface well, but reproduces the precise hydrogen-bonding network and the fine-grained strength of contacts less faithfully.

These population-level trends are illustrated by a representative complex (Fig. 6B–D): AF3 correctly reproduced the interfacial salt bridge together with the majority of non-bonded contacts and interface residues, while incorrectly predicting a subset of the hydrogen bonds present in the experimental structure.

In summary, when AF3 places the antigen and antibody in approximately the correct relative orientation, it reconstructs the binding interface at the residue level with high fidelity—faithfully reproducing epitope and paratope residues, salt bridges, and non-bonded contacts—but with lower accuracy for hydrogen bonds and for the precise strength of individual contacts. AF3 is therefore well suited to identifying epitope and paratope regions in correctly docked complexes, but should be used with caution when atomic-level interaction detail is required.

## 4 Discussion

### 4.1 Antibody Fv structure prediction

We compared three representative antibody structure-prediction frameworks—IgFold (Ruffolo *et al*. 2023), ABB2 (Abanades *et al*. 2023), and AlphaFold3 (AF3) (Abramson *et al*. 2024)—across global Fv architecture and CDR-H3 loop accuracy. All three methods reconstructed the Fv region with high accuracy, but AF3 was the most accurate at both the global Fv level and, notably, the highly variable CDR-H3 loop, and it showed the strongest coupling between global and local accuracy. The two antibody-specific tools were essentially deterministic across repeated predictions, whereas AF3 exhibited modestly higher run-to-run variability. In absolute terms, however, this variability was small and the maximum per-antibody deviations were comparable across all three methods. These results suggest that, among the tools evaluated here, AF3 is the most accurate option for antibody Fv modeling, including CDR-H3 prediction, whereas ABB2 and IgFold remain attractive for high-throughput applications that benefit from fast and highly reproducible predictions.

AF3’s strong performance on CDR-H3 is noteworthy because this hypervariable loop has traditionally been regarded as the regime in which antibody-specific models, with their domain-specific training and antibody-focused inductive biases (Abanades *et al*. 2023, Fernández-Quintero *et al*. 2023, Ruffolo *et al*. 2023, Chen *et al*. 2024), would be expected to hold an advantage. A plausible explanation is that AF3’s substantially larger and more diverse training corpus, deeper architecture, and diffusion-based sampling of conformational space (Ho *et al*. 2020, Abramson *et al*. 2024) together allow it to model CDR-H3 geometries at least as well as specialized tools, although the precise reasons lie beyond the scope of this benchmark. The antibody-specific tools nonetheless remain competitive and, because of their low run-to-run variability, offer a level of reproducibility that is useful in large-scale antibody-modeling workflows.

A persistent, method-independent challenge is that CDR-H3 remains the principal source of Fv structural error for all three tools. Across methods, CDR-H3 deviations were substantially larger and more dispersed than the overall Fv RMSD, and the two were tightly coupled. This reflects the intrinsic flexibility and structural heterogeneity of CDR-H3 and indicates that accurate hypervariable-loop modeling remains a bottleneck even when the overall Fv framework is well reconstructed. Future work integrating the scale and generality of models such as AF3 with antibody-focused training may further improve the robustness of CDR-H3 and other flexible-loop predictions.

### 4.2 Antibody-antigen complex structure prediction

Among the three complex-prediction methods, AF3 (Abramson *et al*. 2024) showed a clear advantage in antibody–antigen complex prediction, with substantially higher interface accuracy as reflected by DockQ (Basu and Wallner 2016) and complex RMSD. It reached at least medium-quality docking for nearly half of the complexes and high-quality docking for a smaller but substantial subset, with successful poses clustering at low complex RMSD. These results indicate that AF3 can recover near-native binding modes for a meaningful subset of antibody–antigen pairs. This finding is also consistent with broader all-atom biomolecular benchmarks showing that antibody–antigen complexes remain among the more challenging interaction classes for current complex predictors (Yin and Pierce 2024, Gaudreault *et al*. 2025, Xu *et al*. 2025).

AF3's advantage is plausibly linked to its general-purpose biomolecular interaction framework and diffusion-based structure-generation process (Ho *et al*. 2020, Abramson *et al*. 2024), which is better suited than rigid-body search to sampling flexible biomolecular interaction geometries. Even so, interface accuracy remained moderate overall, and a large fraction of complexes were still docked incorrectly. This indicates that, although AF3 substantially improves antibody–antigen complex prediction relative to the other tested methods, accurate immune-complex docking remains an open challenge.

In contrast, dyMEAN (Kong *et al*. 2023) and GRAMM (Singh *et al*. 2024) produced few or no medium- or high-quality predictions, highlighting the limitations of epitope-guided modeling and classical rigid-body docking in capturing the flexible and conformationally complex antibody–antigen interface (Desta *et al*. 2020, Kong *et al*. 2023). GRAMM’s consistently poor performance emphasizes that rigid-body docking approaches are insufficient for high-resolution immune-complex prediction when substantial interface flexibility or induced-fit effects are present (Desta *et al*. 2020, Singh *et al*. 2024). Such methods rely heavily on shape complementarity and simplified scoring, whereas antibody–antigen recognition often involves flexible CDR loops, localized conformational adaptation, and fine-grained residue-level complementarity.

The limited performance of dyMEAN in this benchmark also suggests that explicit epitope guidance alone is not sufficient to ensure accurate complex modeling. Although dyMEAN incorporates epitope constraints through shadow-paratope modeling (Kong *et al*. 2023), accurate docking still depends on the quality of the supplied epitope information and on the ability to translate interface constraints into a correct three-dimensional pose. This may explain why an epitope-guided strategy can still underperform a large general-purpose complex predictor when conformational changes and interface geometry are difficult to resolve. Overall, these findings support the growing view that data-driven complex predictors offer clear advantages for antibody–antigen modeling, but also show that current methods still leave substantial room for improvement, especially for targets requiring precise interface placement and flexible-loop accommodation (Yin and Pierce 2024, Gaudreault *et al*. 2025).

An important limitation of the present study is that the benchmark is representative rather than exhaustive. Recently developed frameworks such as DeepSCFold (Hou *et al*. 2025), which uses sequence-derived structural complementarity to construct paired multiple-sequence alignments and has reported competitive antibody–antigen interface prediction relative to AF3, were not included in the predefined experimental framework. Future benchmark updates should evaluate DeepSCFold and other emerging methods using the same target set, input definitions, model-selection criteria, and evaluation metrics, thereby preserving the controlled and statistically consistent design of the present study.

### 4.3 Paratope–epitope interface recovery

Extending the interface analysis from a single representative case to all complexes with reliable docking allowed us to assess how faithfully AF3 reconstructs antibody–antigen binding interfaces in a systematic manner. The central finding is that interface recovery is conditional on docking success: when the predicted pose was approximately correct, AF3 reproduced the binding interface with high fidelity, recovering epitope and paratope residues with high F1 scores, together with a large fraction of residue–residue contacts and interfacial salt bridges. For correctly docked complexes, therefore, AF3 can reliably localize the residues and dominant electrostatic features that define the binding interface.

AF3 was nonetheless less accurate for the finer physicochemical details of molecular recognition. Hydrogen bonds were recovered less frequently than salt bridges or non-bonded contacts, and the contact multiplicity of individual residue pairs, approximated by the number of atom–atom contacts per residue pair extracted by PDBsum (Laskowski 2001), was only moderately correlated with the experimental values. In other words, AF3 reproduced the geometric and topological framework of the interface more accurately than the detailed hydrogen-bonding network or the precise relative weighting of individual contacts.

These limitations are consistent with the structural nature of antibody–antigen binding interfaces. Large-scale analyses of experimentally determined antibody–antigen complexes have shown that immune recognition is shaped by localized CDR–epitope contacts, recurring hotspot residues, and chemically specific residue–residue interactions (Madsen *et al*. 2024). Such features may be more difficult to recover than the overall binding pose, particularly when hydrogen-bond geometry and side-chain packing contribute disproportionately to recognition specificity. Thus, the present results suggest that AF3 can capture the overall interface region in correctly docked cases, but that its predictions of atomic-level interaction detail should be interpreted with caution.

This distinction has practical implications for structure-based antibody engineering. AF3-predicted complexes may be useful for identifying candidate epitope and paratope regions, prioritizing broad interface hypotheses, and guiding downstream experimental inspection when the docking pose is reliable. However, predicted hydrogen bonds, contact multiplicities, and fine-grained energetic interpretations should not be treated as direct substitutes for experimentally resolved interface chemistry. Improving the fidelity of hydrogen-bonding and contact-multiplicity prediction will likely require more antibody–antigen-specific structural data, interface-aware training objectives, or post-prediction refinement strategies that explicitly account for side-chain packing and local interaction chemistry.

In summary, AF3 is a strong tool for identifying epitope and paratope regions in correctly docked antibody–antigen complexes, but its predictions of atomic-level interaction detail remain less reliable than its recovery of the overall interface topology. Because interface recovery depends first on obtaining a correct docking pose, improvements in docking accuracy and in interface chemistry are complementary priorities for the next generation of immune-complex structure predictors.

## 5 Conclusion

This study evaluates current publicly available computational tools for antibody structure prediction and antigen–antibody complex modeling. By benchmarking ABodyBuilder2, IgFold, dyMEAN, GRAMM, and AlphaFold3 (AF3) on a non-redundant set of 50 complexes with paired statistical testing, we characterized their performance across three levels: antibody Fv accuracy, antigen–antibody docking quality, and paratope–epitope interface recovery.

AF3 was the most accurate method across all three levels. It achieved the best Fv accuracy, including for the CDR-H3 loop, and clearly outperformed the other methods in antigen–antibody docking, reaching at least medium-quality binding poses (DockQ ≥ 0.49) for 46% of complexes and recovering epitope and paratope residues and salt bridges with high fidelity when docking succeeded. However, AF3 still showed limited accuracy for fine-scale features such as hydrogen-bonding networks and contact strengths, and roughly half of the complexes were docked incorrectly—indicating that the model captures geometric complementarity well but does not yet reproduce the complete energetic landscape of antigen–antibody recognition.

Traditional rigid-body docking (GRAMM) and the geometry-driven dyMEAN showed markedly lower interface accuracy, underscoring the advantage of large-scale data-driven architectures for generalizing to unseen complexes. The comparison also highlights two persistent challenges for high-fidelity, structure-based antibody design: the accuracy of CDR-H3 modeling and the reconstruction of conformational detail at the antigen–antibody interface.

Future work should aim to (1) incorporate antibody-specific and immune-complex–focused training to improve CDR-loop and interface accuracy; (2) couple deep-learning structure prediction with explicit energetic or dynamic constraints to improve biophysical realism; and (3) develop and periodically update unified benchmarks that jointly assess structure prediction, docking, and interface recovery, including recently developed methods such as DeepSCFold under the same controlled evaluation framework.

Overall, this work offers a controlled, statistically grounded comparison of current antibody-modeling tools and provides practical guidance for tool selection in antibody-engineering workflows.

## Supplementary Material

vbag223_Supplementary_Data

## Data Availability

The data and code underlying this article are openly available in Zenodo, at https://zenodo.org/records/20710876. The repository contains the experimental reference structures (split into Fv and antigen regions), all model predictions (five seeds per target for ABodyBuilder2, IgFold, and AlphaFold3 for Fv structure prediction, and for GRAMM, dyMEAN, and AlphaFold3 for complex prediction), the per-target evaluation metrics (TM-score, lDDT, DockQ, RMSD, and CDR-H3 RMSD), the clustering, interface, and statistical-analysis outputs, and the visualization code.

## References

[vbag223-B1] Abanades B , Georges G, Bujotzek A et al ABlooper: fast accurate antibody CDR loop structure prediction with accuracy estimation. Bioinformatics 2022;38:1877–80. 10.1093/bioinformatics/btac016. 35099535 PMC8963302

[vbag223-B2] Abanades B , Wong WK, Boyles F et al ImmuneBuilder: Deep-Learning models for predicting the structures of immune proteins. Commun Biol 2023;6:575. 10.1038/s42003-023-04927-7. 37248282 PMC10227038

[vbag223-B3] Abramson J , Adler J, Dunger J et al Accurate structure prediction of biomolecular interactions with AlphaFold 3. Nature 2024;630:493–500. 10.1038/s41586-024-07487-w. 38718835 PMC11168924

[vbag223-B4] Basu S , Wallner B. DockQ: a quality measure for protein-protein docking models. PLoS One 2016;11:e0161879. 10.1371/journal.pone.0161879. 27560519 PMC4999177

[vbag223-B5] Benjamini Y , Hochberg Y. Controlling the false discovery rate: a practical and powerful approach to multiple testing. R Stat Soc J Ser B Methodol 1995;57:289–300. 10.1111/j.2517-6161.1995.tb02031.x.

[vbag223-B6] Berman HM , Westbrook J, Feng Z et al The Protein Data Bank. Nucleic Acids Res 2000;28:235–42. 10.1093/nar/28.1.235. 10592235 PMC102472

[vbag223-B7] Chan AC , Carter PJ. Therapeutic antibodies for autoimmunity and inflammation. Nat Rev Immunol 2010;10:301–16. 10.1038/nri2761. 20414204

[vbag223-B8] Chen H , Fan X, Zhu S et al Accurate prediction of CDR-H3 loop structures of antibodies with deep learning. eLife 2024;12:RP91512. 10.7554/eLife.91512. 38921957 PMC11208048

[vbag223-B9] Chiu ML , Goulet DR, Teplyakov A et al Antibody structure and function: the basis for engineering therapeutics. Antibodies (Basel) 2019;8:55. 10.3390/antib8040055. 31816964 PMC6963682

[vbag223-B10] Desta IT , Porter KA, Xia B et al Performance and its limits in rigid body Protein-Protein docking. Structure 2020;28:1071–81.e3. 10.1016/j.str.2020.06.006. 32649857 PMC7484347

[vbag223-B11] Escobedo N , Saldaño T, Mac Donagh J et al Revealing missing protein–ligand interactions using AlphaFold predictions. J Mol Biol 2024;436:168852. 10.1016/j.jmb.2024.168852. 39510344

[vbag223-B12] Evans R , O’Neill M, Pritzel A et al Protein complex prediction with AlphaFold-multimer. bioRxiv. 2021. 10.1101/2021.10.04.463034.

[vbag223-B13] Fernández-Quintero ML , Kokot J, Waibl F et al Challenges in antibody structure prediction. MAbs 2023;15:2175319. 10.1080/19420862.2023.2175319. 36775843 PMC9928471

[vbag223-B14] Gaudreault F , Sulea T, Corbeil CR. AI-augmented physics-based docking for antibody-antigen complex prediction. Bioinformatics 2025;41:1–9. 10.1093/bioinformatics/btaf129. PMC11978387 40135432

[vbag223-B15] He XH , Li JR, Shen SY et al AlphaFold3 versus experimental structures: assessment of the accuracy in ligand-bound G protein-coupled receptors. Acta Pharmacol Sin 2025;46:1111–22. 10.1038/s41401-024-01429-y. 39643640 PMC11950431

[vbag223-B16] Ho J , Jain A, Abbeel P. Denoising diffusion probabilistic models. Adv Neural Inform Proces Syst 2020;33:6840–51. https://proceedings.neurips.cc/paper_files/paper/2020/hash/4c5bcfec8584af0d967f1ab10179ca4b-Abstract.html (5 August 2026, date last accessed).

[vbag223-B17] Hou M , Xia Y, Wang P et al High-accuracy protein complex structure modeling based on sequence-derived structure complementarity. Nat Commun 2025;16:10182. 10.1038/s41467-025-65090-7. 41261173 PMC12630834

[vbag223-B18] Joubbi S , Micheli A, Milazzo P et al Antibody design using deep learning: from sequence and structure design to affinity maturation. Brief Bioinform 2024;25:bbae307. 10.1093/bib/bbae307. 38960409 PMC11221890

[vbag223-B19] Jumper J , Evans R, Pritzel A et al Highly accurate protein structure prediction with AlphaFold. Nature 2021;596:583–9. 10.1038/s41586-021-03819-2. 34265844 PMC8371605

[vbag223-B20] Kabsch W. A solution for the best rotation to relate two sets of vectors. Acta Cryst A 1976;32:922–3. 10.1107/S0567739476001873.

[vbag223-B21] Kong X , Huang W, Liu Y. End-to-end full-atom antibody design. In: *Proceedings of the 40th International Conference on Machine Learning (ICML), PMLR 2023*, Vol. 202, p. 17409–29. 10.48550/arXiv.2302.00203.

[vbag223-B22] Laskowski RA. PDBsum: summaries and analyses of PDB structures. Nucleic Acids Res 2001;29:221–2. 10.1093/nar/29.1.221. 11125097 PMC29784

[vbag223-B23] Leem J , Dunbar J, Georges G et al ABodyBuilder: automated antibody structure prediction with data–driven accuracy estimation. MAbs 2016;8:1259–68. 10.1080/19420862.2016.1205773. 27392298 PMC5058620

[vbag223-B24] Madsen AV , Mejias-Gomez O, Pedersen LE et al Structural trends in antibody-antigen binding interfaces: a computational analysis of 1833 experimentally determined 3D structures. Comput Struct Biotechnol J 2024;23:199–211. 10.1016/j.csbj.2023.11.056. 38161735 PMC10755492

[vbag223-B25] Mariani V , Biasini M, Barbato A et al lDDT: a local superposition-free score for comparing protein structures and models using distance difference tests. Bioinformatics 2013;29:2722–8. 10.1093/bioinformatics/btt473. 23986568 PMC3799472

[vbag223-B26] Pei J , Andreeva A, Chuguransky S et al Bridging the gap between sequence and structure classifications of proteins with AlphaFold models. J Mol Biol 2024;436:168764. 10.1016/j.jmb.2024.168764. 39197652 PMC13126517

[vbag223-B27] Ruffolo JA , Chu LS, Mahajan SP et al Fast, accurate antibody structure prediction from deep learning on massive set of natural antibodies. Nat Commun 2023;14:2389. 10.1038/s41467-023-38063-x. 37185622 PMC10129313

[vbag223-B28] Ruffolo JA , Gray JJ, Sulam J. Deciphering antibody affinity maturation with language models and weakly supervised learning. arXiv, 10.48550/arXiv.2112.07782, 14 December 2021, preprint: not peer reviewed.

[vbag223-B29] Sanaboyana VR , Elcock AH. Improving signal and transit peptide predictions using AlphaFold2-predicted protein structures. J Mol Biol 2024;436:168393. 10.1016/j.jmb.2023.168393. 38065275 PMC10843742

[vbag223-B30] Santuari L , Bachmann Salvy M, Xenarios I et al AI-accelerated therapeutic antibody development: practical insights. Front Drug Discov 2024;4:1447867. 10.3389/fddsv.2024.1447867.

[vbag223-B31] Satorras VG , Hoogeboom E, Welling M. E(n) Equivariant Graph Neural Networks. In: Proceedings of the 38th International Conference on Machine Learning, p. 9323–32. Cambridge, MA: PMLR, 2021. https://proceedings.mlr.press/v139/satorras21a.html (5 August 2026, date last accessed).

[vbag223-B32] Schrödinger, LLC. The PyMOL Molecular Graphics System, version 3.05. 2024.

[vbag223-B33] Singh A , Copeland MM, Kundrotas PJ et al GRAMM web server for protein docking. In: Gore M, Jagtap UB (eds), Computational Drug Discovery and Design. New York, NY: Springer US, 2024, 101–12. 10.1007/978-1-0716-3441-7_5. 37676594

[vbag223-B34] Sliwkowski MX , Mellman I. Antibody therapeutics in cancer. Science 2013;341:1192–8. 10.1126/science.1241145. 24031011

[vbag223-B35] Steinegger M , Söding J. MMseqs2 enables sensitive protein sequence searching for the analysis of massive data sets. Nat Biotechnol 2017;35:1026–8. 10.1038/nbt.3988. 29035372

[vbag223-B36] Swindells MB , Porter CT, Couch M et al abYsis: integrated antibody sequence and structure—management, analysis, and prediction. J Mol Biol 2017;429:356–64. 10.1016/j.jmb.2016.08.019. 27561707

[vbag223-B37] Waterhouse AM , Studer G, Robin X et al The structure assessment web server: for proteins, complexes and more. Nucleic Acids Res 2024;52:W318–23. 10.1093/nar/gkae270. 38634802 PMC11223858

[vbag223-B38] Xu S , Feng Q, Qiao L et al Benchmarking all-atom biomolecular structure prediction with FoldBench. Nat Commun 2025;17:442. 10.1038/s41467-025-67127-3. 41345395 PMC12800276

[vbag223-B39] Yan Y , Tao H, He J et al The HDOCK server for integrated protein–protein docking. Nat Protoc 2020;15:1829–52. 10.1038/s41596-020-0312-x. 32269383

[vbag223-B40] Yin R , Pierce BG. Evaluation of AlphaFold antibody–antigen modeling with implications for improving predictive accuracy. Protein Sci 2024;33:e4865. 10.1002/pro.4865. 38073135 PMC10751731

[vbag223-B41] Zhang Y , Skolnick J. Scoring function for automated assessment of protein structure template quality. Proteins 2004;57:702–10. 10.1002/prot.20264. 15476259

