# Supplementary material for: Benchmarking antibody modeling tools across structure prediction, docking, and paratope–epitope interface analysis
Source: Bioinform Adv. 2026 Aug 11;6(1):vbag223. doi: 10.1093/bioadv/vbag223 (PMC13557113; doi:10.1093/bioadv/vbag223)
Supplement: Supplementary file 1 [file vbag223_supplementary_data.zip › Supplementary.docx]

| number_of_sequences | number_of_clusters | largest_cluster_size | singleton_clusters | clustering_level | identity_cutoff |
| --- | --- | --- | --- | --- | --- |
| 50 | 49 | 2 | 48 | heavy | 0.9 |
| 50 | 50 | 1 | 50 | heavy | 0.95 |
| 50 | 43 | 5 | 39 | light | 0.9 |
| 50 | 46 | 5 | 45 | light | 0.95 |
| 50 | 49 | 2 | 48 | fv_concat | 0.9 |
| 50 | 50 | 1 | 50 | fv_concat | 0.95 |
| 50 | 50 | 1 | 50 | cdrh3 | 0.9 |
| 50 | 50 | 1 | 50 | cdrh3 | 0.95 |

Table S1. Summary clustering results. Full cluster membership information is provided in Supplementary Data 2.

| Tool | Task | Deployment | Version / access date | Seed & sampling strategy | Key parameters | Hardware / server | Approximate cost |
| --- | --- | --- | --- | --- | --- | --- | --- |
| ABB2 | Fv prediction | Local | GitHub repository snapshot; commit <0df4e2a>, Nov 8, 2024 | Repeated local runs with 5 different random seeds | Default parameters | 4 × RTX 3080 10 GB | ~80 s / 5 outputs per target |
| IgFold | Fv prediction | Local | GitHub repository snapshot; commit <85d9dcd>, Sep 3, 2023 | Repeated local runs with 5 different random seeds | do_refine=True; do_renum=True | 4 × RTX 3080 10 GB | ~90 s / 5 outputs per target |
| AlphaFold3 | Fv prediction & Complex prediction | Web server | Accessed Sep 2025; official AlphaFold3 server | Five server-generated models returned by the AF3 server | default web-server settings | official AF3 web server | ~3–5 min / 5 outputs per target |
| GRAMM | Complex docking | Web server | Accessed Sep 2025; public GRAMM web server | Top five docking poses returned by the web server | default web-server settings; top five returned poses retained | public GRAMM web server | ~2–3 min / 5 outputs per target |
| dyMEAN | Complex modeling | Local | GitHub repository snapshot; commit <ecfdfa8>, Apr 18, 2024 | Local runs with parameters n_sample = 5 | n_sample=5; enable_openmm_relax=True | 4 × RTX 3080 10 GB | ~135 s / 5 outputs per target |

Table S2. Details for each tool.

| Tool | Fv RMSD — median per-target SD (Å) | CDR-H3 RMSD — median per-target SD (Å) |
| --- | --- | --- |
| ABB2 | 0.003 | 0.016 |
| IgFold | 0.002 | 0.008 |
| AF3 | 0.064 | 0.144 |

Table S3. Within-tool run-to-run variability. For each Fv predictor, the per-target standard deviation across the five predictions, summarized as the median across the 50 targets. The two antibody-specific tools are essentially deterministic, whereas AF3 shows higher but still small variability; in all cases the run-to-run SD is far smaller than the between-tool differences (Table S4).

| Fv-task metric | ABB2 vs IgFold | ABB2 vs AF3 | IgFold vs AF3 |
| --- | --- | --- | --- |
| TM-score | 0.053 | 0.002 | <0.001 |
| lDDT | 0.049 | <0.001 | <0.001 |
| DockQ | 0.002 | 0.001 | <0.001 |
| CDR-H3 RMSD | 0.752 | 0.026 | 0.017 |

| Complex-task metric | GRAMM vs dyMEAN | GRAMM vs AF3 | dyMEAN vs AF3 |
| --- | --- | --- | --- |
| TM-score | <0.001 | <0.001 | <0.001 |
| lDDT | <0.001 | <0.001 | <0.001 |
| DockQ (Ag–H) | <0.001 | <0.001 | <0.001 |
| DockQ (Ag–L) | <0.001 | <0.001 | <0.001 |
| Complex RMSD | <0.001 | <0.001 | <0.001 |

Table S4. Between-tool pairwise statistical comparisons. Benjamini–Hochberg-adjusted p-values from two-sided Wilcoxon signed-rank tests on paired per-target values (n = 50). Fv-level metrics compare the three Fv predictors; complex-level metrics compare the three complex predictors.

| Metric | All (n=50) | DockQ ≥ 0.23 (n=27) | DockQ ≥ 0.49 (n=23) |
| --- | --- | --- | --- |
| Epitope precision | 0.591 (0.497–0.685) | 0.851 (0.811–0.888) | 0.870 (0.835–0.903) |
| Epitope recall | 0.585 (0.487–0.679) | 0.848 (0.804–0.891) | 0.876 (0.835–0.915) |
| Epitope F1 | 0.582 (0.486–0.673) | 0.843 (0.809–0.874) | 0.866 (0.841–0.891) |
| Paratope precision | 0.695 (0.633–0.753) | 0.837 (0.800–0.873) | 0.848 (0.809–0.884) |
| Paratope recall | 0.683 (0.617–0.744) | 0.837 (0.791–0.879) | 0.867 (0.828–0.903) |
| Paratope F1 | 0.683 (0.620–0.739) | 0.832 (0.796–0.866) | 0.853 (0.820–0.883) |
| Paratope VH F1 | 0.708 (0.650–0.764) | 0.844 (0.806–0.879) | 0.856 (0.813–0.897) |
| Paratope VL F1 | 0.601 (0.521–0.680) | 0.785 (0.722–0.842) | 0.819 (0.765–0.869) |
| Contact-pair precision | 0.399 (0.302–0.497) | 0.702 (0.637–0.760) | 0.743 (0.696–0.788) |
| Contact-pair recall | 0.392 (0.294–0.491) | 0.698 (0.624–0.762) | 0.753 (0.703–0.799) |
| Contact-pair F1 | 0.391 (0.298–0.488) | 0.694 (0.627–0.751) | 0.742 (0.700–0.780) |
| Salt-bridge recall | 0.427 (0.294–0.566) | 0.812 (0.701–0.912) | 0.840 (0.731–0.936) |
| Non-bonded recall | 0.392 (0.293–0.491) | 0.697 (0.623–0.760) | 0.753 (0.705–0.798) |
| H-bond recall | 0.323 (0.234–0.410) | 0.581 (0.503–0.656) | 0.639 (0.577–0.701) |
| Contact-strength Spearman | 0.470 (0.385–0.551) | 0.549 (0.480–0.615) | 0.573 (0.498–0.643) |

Table S5. Paratope–epitope interface recovery by AlphaFold3 across docking-quality strata. Mean (bootstrap 95% CI) of AF3 interface recovery relative to the experimental interface, for all 50 complexes and for the subsets with DockQ ≥ 0.23 (n = 27) and DockQ ≥ 0.49 (n = 23). Salt-bridge recall is computed only over complexes whose reference interface contains interfacial salt bridges (n = 39, 20, and 17 for the three columns, respectively).
